# Extraction and Characterization of Aceh Bovine Bone-Derived Hydroxyapatite for Applications in Dentistry

**DOI:** 10.1055/s-0045-1802946

**Published:** 2025-03-12

**Authors:** Viona Diansari, Rinaldi Idroes, Sunarso Sunarso, Sri Fitriyani

**Affiliations:** 1 Graduate School of Mathematics and Applied Sciences, Universitas Syiah Kuala, Banda Aceh, Indonesia; 2 Department of Dental Material, Faculty of Dentistry, Universitas Syiah Kuala, Banda Aceh, Indonesia; 3 Department of Pharmacy, Faculty of Mathematics and Natural Sciences, Universitas Syiah Kuala, Banda Aceh, Indonesia; 4 Department of Dental Material, Faculty of Dentistry, Universitas Indonesia, Jakarta, Indonesia

**Keywords:** hydroxyapatite, Aceh bovine bone, calcination, Fourier transform infrared (FTIR), X-ray diffraction (XRD), scanning electron microscope–energy dispersive X-ray (SEM-EDX)

## Abstract

**Objective:**

Bone grafts derived from natural hydroxyapatite (HA) are increasingly being explored because they are more economical in terms of production costs compared with commercial HA. HA can be obtained from local cattle slaughter waste in Aceh, Indonesia, which has not been widely studied for its potential for dental applications. This study examines the synthesis and characterization of bovine HA (BHA) derived from Aceh cattle femur through calcination for applications in dentistry.

**Materials and Methods:**

This research began with the cleaning of fresh bones by boiling and soaking them in acetone for 2 hours before 3-hour calcination at varying temperatures. The BHA samples were characterized using Fourier transform infrared (FTIR) spectroscopy, X-ray diffraction, scanning electron microscopy with energy dispersive X-ray (SEM-EDX), and particle size analyzer (PSA).

**Statistical Analysis:**

Data were analyzed using SPSS with a one-way analysis of variance to assess the impact of calcination temperature on the yield and particle size of BHA.

**Results:**

BHA obtained from calcination at 900°C and 1,000°C showed the highest crystallinity, with values above 84%, and uniform particle distribution. PSA and SEM analysis showed that BHA particles were spherical in submicron size, which became smaller and more uniform but agglomeration did not occur significantly between each increase in calcination temperature. FTIR analysis showed the presence of phosphate, carbonate, and hydroxyl functional groups. Elemental composition analysis using EDX confirmed that essential elements such as calcium and phosphorus were distributed consistently at all temperatures with a Ca/P ratio of 1.7 to 2.3.

**Discussion:**

Based on the characteristics of crystallinity, particle size, and chemical composition of the obtained BHA, it is considered optimal for bioactivity, which allows stimulation of new bone tissue formation and promotes osseointegration while balancing structural stability. This makes BHA derived from Aceh cattle bones a suitable bone filler candidate for treating alveolar bone defects in hard tissue regeneration. These findings highlight the potential use of cattle bone waste as a sustainable source of HA in dental applications.

**Conclusion:**

These findings suggest that Aceh bovine bones are a viable source for producing quality BHA, potentially contributing to more sustainable and ecofriendly biomaterials for dental applications.

## Introduction

Musculoskeletal issues impact nearly 1.71 billion people worldwide, 1 it is vital to have a reliable and effective way to treat these conditions. To address bone defects such as osteoporosis, calcium deficiency issues, and injuries like fractures, the use of hydroxyapatite (HA) in treatment is necessary. 2 HA has attracted significant interest for biomedical applications due to its excellent compatibility with body tissues, making it one of the most widely developed biomaterials for bone implants. 3 4 HA also enhances osteoblast adhesion and viability, and can interact with living bone tissue without causing cytotoxicity or an inflammatory response. 2 5 6 7

The use of HA in dentistry has gained significant attention over the last decade due to its excellent biocompatibility and similarity to natural bone minerals making HA ideal for applications in oral implant and bone reconstruction, as well in restorative and preventive dental treatment. 8 HA can be synthesized artificially by reacting phosphoric acid with calcium hydroxide or isolated from various natural sources, both inorganic and organic. Inorganic sources include limestone and phosphate-containing rocks, while organic sources can come from seashells, coral, eggshells, red algae, fish bones and scales (such as tuna, catfish, tilapia, cod, squid), and mammalian bones, like those of cows and pigs. 4 9 The usage of HA extracted from natural sources is an environmentally friendly, sustainable, and cost-effective method to produce this material, given their abundant availability. This approach can positively impact the economy, environment, and overall public health.

Bovine HA (BHA) is an inorganic material derived from bovine bones, used as an alternative composite component. It is composed of 93% HA (Ca _10_ (PO _4_ ) _6_ (OH) _2_ ) and 7% β-tricalcium phosphate (Ca _3_ (PO _4_ ) _2_ ). 10 Other studies report that bovine bones are chosen as a source of natural BHA due to their high calcium content, approximately 85.84%, as well as their composition of 57% HA and 33% organic material. 5 9 Aceh Province, one of the local bovine producers in Indonesia, typically raises cattle from crosses between *Bos indicus* and *Bos sondaicus* or *Bos javanicus* . There is a high consumption of bovine meat, particularly during traditional events such as *kenduri* (weddings, *Aqiqah* ) and religious celebrations (celebration of the birth of Prophet Muhammad, Eid al-Fitr, and Eid al-Adha). Aceh's culture, closely tied to traditional ceremonies, results in a higher amount of bovine bone waste compared with other regions. The size of Aceh bovine is considered medium, influenced by age and sex, with a weight of 29 to 30 kg and a bone weight of 16%. Aceh bovine are known for their resilience to environmental conditions, disease resistance, and high fertility. 11 An analysis effectively grouped the bone samples into three distinct categories (Aceh, Bali, and Brahman), clearly showing the close similarity between the two Aceh bovine bones, as they were grouped together. 12 However, differences in the composition ratio may still occur. Nevertheless, there have not been many studies conducted on the characteristics of Aceh bovine that could influence the characterization of BHA. In particular, the exploration of the potential of Aceh bovine-derived HA for medical and dental applications has yet to be undertaken.

BHA, particularly when derived from bone waste, presents a sustainable and economical alternative for dental applications, such as bone grafts and dental implants. In this context, bovine bone-derived HA not only minimizes waste but also provides a high-purity source of calcium phosphate, which can enhance osseointegration in dental procedures. The development of BHA from Aceh bovine bones, known for their high calcium content, could therefore be highly valuable for advancing materials used in dental restorations.

HA can be synthesized using a variety of techniques. These methods are broadly categorized into dry techniques—such as solid-state synthesis and mechanochemical processes—and wet techniques, which include chemical precipitation, hydrolysis, sol-gel methods, hydrothermal synthesis, emulsion techniques, and sonochemical processes. 3 Compared with other methods, dry calcination is a simple technique for extracting BHA that effectively eliminates germs and impurities without compromising the bioactivity of BHA. 2 13 Calcination temperature has a crucial impact on the formation of BHA. 14 This study aims to extract BHA from Aceh bovine bones using various calcination temperatures. This synthesis method allows the thermal decomposition of organic components in bones, effectively eliminating them, while also removing any genomic traces of diseases, ensuring high biological safety. 5 The impact of calcination temperature on the use of Aceh bovine bones as a source of natural BHA has not yet been investigated. The extracted BHA will be characterized using Fourier-transform infrared (FTIR) spectroscopy, particle size analyzer (PSA), X-ray diffractometer (XRD), and scanning electron microscopy (SEM) with energy dispersive X-ray (EDX) techniques.

## Materials and Methods

### Materials

The materials used in this research were ethanol 96% (technical grade) and acetone pro-analysis (Sigma-Aldrich, Steinheim, Germany). Another ingredient is distilled water.

### Procedures

**Fig. 1:**
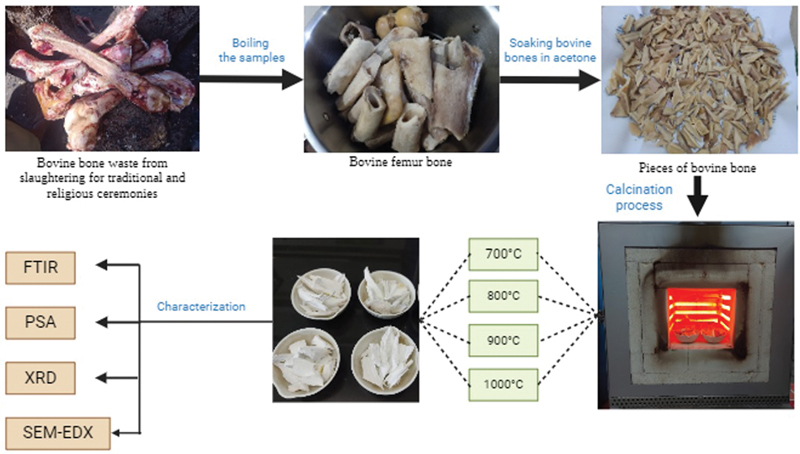
Illustration of the research procedure.

This study is an initial investigation with a limited scope, focusing on only three main stages: sample preparation, calcination, and characterization ( Fig. 1 ).

The methodology of this study was based on the Indonesian National Standard, which is adopted from International Organization for Standardization (ISO) 13779 for testing HA powders in medical applications, including dental surgery implants. In this preliminary research on BHA derived from Aceh bovine bones, physicochemical characterization tests were conducted in accordance with the aforementioned ISO standard. These tests included FTIR to identify chemical functional groups, XRD to assess crystallinity, SEM to analyze particle morphology and distribution, and EDX to determine the chemical composition. Particle size analysis (granulometry) was performed quantitatively using PSA.

### Bovine Bone Preparation

The Aceh bovine bones were collected from the Lambaro slaughter house at traditional market in Aceh Besar Regency, Aceh Province, Indonesia, as waste from beef processing. The selected bone samples include the following criteria, specifically femur bones from male cattle aged 2 to 3 years. It is known that male Aceh cattle have larger body sizes compared with female Aceh cattle. 11 The bovine bones were boiled in water maintained at a rolling boil for 2 hours to remove fat and help eliminate macroimpurities. This procedure has been verified to effectively cleanse the bones. They were then washed and cleaned with distilled water to remove any remaining meat, tendons, bone marrow, and other soft tissues. To maximize the elimination of residual organic material, the samples were soaked in ethanol 96% for 8 hours. After rinsing with distilled water, the bones were cut into small pieces (3–4 cm) using a bone cutter and soaked in acetone solution for 2 hours. 15 Acetone was used to remove any residual invisible fat from the bones. 16 The bovine bones were rinsed twice with deionized water, then dried under sunlight for 6 to 8 hours, and air-dried for an additional week to ensure the BHA samples were fully dry before weighing for the calcination process.

### Calcination

The dried bone pieces, free of fat tissue, were first weighed. Calcination was carried out at varying temperatures of 700, 800, 900, and 1,000°C for 3 hours using a furnace (Barnstead Thermolyne Furnance, Massachusetts, United States) for 100 g of each bone sample. After the furnace temperature had dropped to a level safe for opening, the bones were placed in a drying oven (Drying Oven UN-55 Memmert, Schwabach, Germany) at 100° C for 1 hour to prevent sudden temperature fluctuations upon removal from the furnace. The BHA samples were then allowed to cool to room temperature in a porcelain dish. Afterward, the BHA samples were ground into a fine powder using a mortar and pestle, along with a grinding tool (Dry Grinder-3000W, Huachuan, China). This bone powder was then sieved using a sieve shaker (Sieve Shaker AS200, Haan, Germany) with a 140 mesh size, resulting in HA powder with particle sizes less than 100 µm. All work procedures are performed with three repetitions. The powder was weighed and stored in a sealed container and placed in a desiccator to prevent contamination by air and moisture before undergoing characterization to confirm the obtained HA.

### Characterization

All samples in this study were characterized using various instruments according to ISO 13779 for characterization testing. The functional group characteristics of the HA powder were analyzed using FTIR (Shimadzu IRPrestige-21 FTIR spectrophotometer, Kyoto, Japan) with a sample-to-KBr ratio of 1:10 (4000–1000/cm). Particle size distribution was examined using a PSA (Shimadzu SALD-2300, Kyoto, Japan). The crystallinity of the BHA samples was assessed using an XRD (XRD-700, Kyoto Japan). The morphology, particle gap, and chemical composition of the BHA samples were analyzed using SEM-EDX (Thermo Scientific Type Prisma E, Massachusetts, United States).

The data analysis was performed using Statistical Product and Service Solution (SPSS) software, applying a parametric one-way analysis of variance test to determine the effect of variations in calcination temperature on BHA obtained from Aceh bovine bones (yield) and its particle size.

## Results

### BHA Preparation

**Fig. 2:**
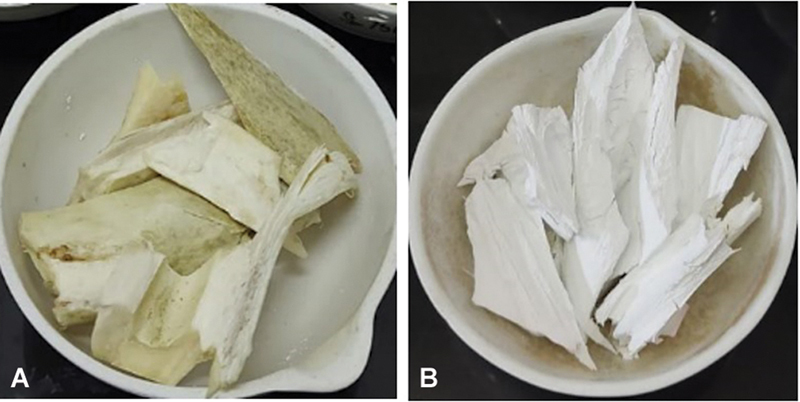
Bovine bone before ( **A** ) and after ( **B** ) calcination.

The bovine bone color changed from white before calcination to a white hue after calcination, indicating the successful formation of natural BHA ( Fig. 2 ). Based on the calculation of the difference between the dry bone weight and the obtained BHA powder weight, the yield percentage was determined ( Table 1 ).

**Table 1:** Percentage yield of BHA

Calcination temperature (°C)	Weight, mean ± SD (g)		Percentage yield, mean ± SD (%)	Significance
	Bovine bones	Bovine hydroxyapatite		
700	100 ± 0.000	55.62 ± 0. 0028	52.62 ± 0.003	0.000
800	100 ± 0.000	59.56 ± 0.0007	59.56 ± 0.001	
900	100 ± 0.000	60.54 ± 0. 0014	60.54 ± 0.001	
1,000	100 ± 0.000	59.80 ± 0.0020	59.80 ± 0.002	

Abbreviations: BHA, bovine hydroxyapatite; SD, standard deviation.

### FTIR Analysis

**Fig. 3:**
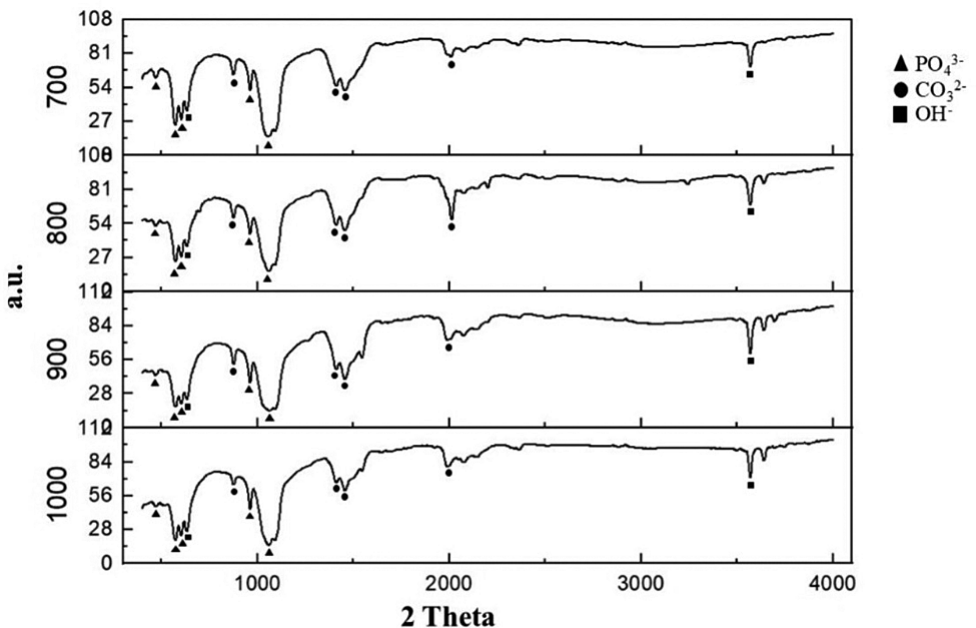
Fourier-transform infrared (FTIR) spectra of the bovine hydroxyapatite (BHA).

The identifying functional group of the BHA is shown in Fig. 3 . The BHA exhibits the characteristic compounds of HA, including the chemical functional groups phosphate (PO _4_^3−^ ), carbonate (CO _3_^2−^ ), and hydroxyl (OH ^−^ ). The detailed wave frequencies of the FTIR absorption bands are presented in Table 2 .

**Table 2:** FTIR wave frequencies of BHA

Functional group	Calcination temperature				Vibration model
	700°C	800°C	900°C	1,000°C	
Phosphate, PO _4_^3-^	470.63 572.86 603.72 962.48 1055.06 1093.64	470.63 572.86 603.72 962.48 1058.92 1089.78	468.70 572.86 603.72 962.48 1062.78 1089.78	468.70 572.86 603.72 962.48 1058.92 1091.71	υ _2_ symmetric bending υ _4_ asymmetric bending υ _4_ asymmetric bending υ _1_ symmetric stretching υ _3_ asymmetric stretching υ _3_ asymmetric stretching
Carbonate, CO _3_^2-^	875.68 1411.89 1456.26	875.68 1411.89 1458.18	875.68 1411.89 1458.18 1990.54	875.68 1413.82 1458.18 1990.54	asymmetric bending asymmetric stretching asymmetric stretching asymmetric stretching
Hydroxyl, OH ^-^	632.65 3570.24	632.65 3570.24	632.65 3570.24	632.65 3570.24	asymmetric stretching asymmetric stretching

Abbreviations: BHA, bovine hydroxyapatite; FTIR, Fourier-transform infrared.

### PSA Characterization

The PSA is an instrument used to measure the particle size of a sample and determine its size distribution. This tool operates using the dynamic light scattering method with a maximum wavelength of 633 nm. The particle size distribution indicates the percentage of particles within a specific size range (or interval), known as size classes or fractions. Percentiles are denoted by the letter “d” followed by a percentage value, as shown in Table 3 . For example, D50% = 38.165 µm means that 50% of the BHA sample particles are smaller than 38.165 µm. The D50% value, also referred to as the “median,” divides the particle size distribution into two equal parts—one with particles smaller than the median and the other with particles larger than the median. The particle size distribution of natural BHA from Aceh bovine bones is presented in Table 3 . The particle sizes of the BHA in this study are in the micron range.

**Table 3:** Particle size distribution of BHA

Temperature (°C)	Particle size distribution (µm)			D modal (µm)	D, mean ± SD (µm)	*p*
	D _25%_	D _50%_	D _75%_			
700	6.311	38.165	80.279	79.317	21.332 ± 0.738	1.000
800	23.010	59.191	97.606	89.339	31.653 ± 0.738	
900	7.743	78.588	192.572	89.339	38.519 ± 0.908	
1,000	29.590	62.574	103.173	79.317	38.436 ± 0.700	

Abbreviations: BHA, bovine hydroxyapatite; SD, standard deviation.

### XRD Results

**Fig. 4:**
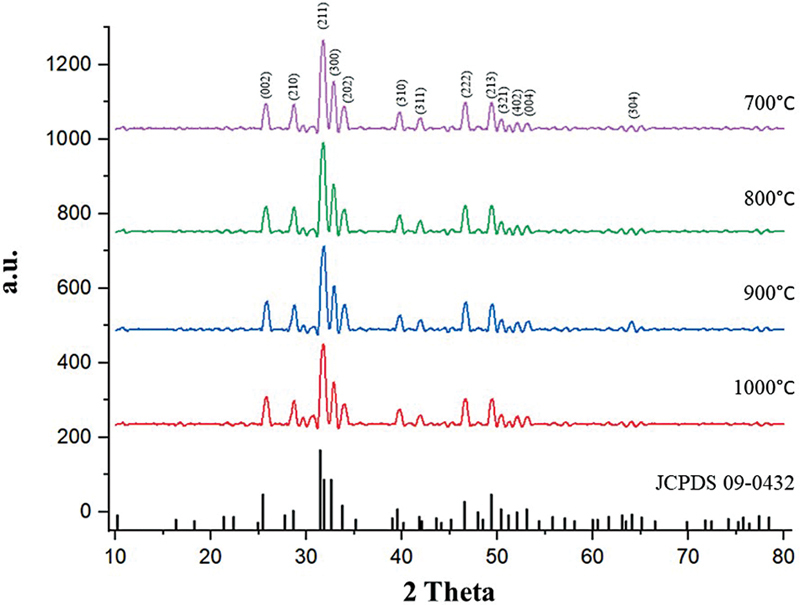
X-ray diffractometer (XRD) spectra of bovine hydroxyapatite (BHA) compared with hydroxyapatite (HA) standard.

The provided XRD spectra show main diffraction peaks identified at approximately 25.9°, 31.8°, 32.9°, 34.1°, 39.8°, 46.7°, 49.4°, and 53.3°, corresponding to the crystallographic planes (002), (211), (112), (300), (310), (222), (213), and (004) of HA with a hexagonal crystal structure, based on the Joint Committee on Powder Diffraction Standards database card number 09-0432 ( Fig. 4 ).

The percentage crystallinity of the natural BHA obtained can be calculated using the following equation:



The calculation results for the crystallinity of natural HA from Aceh bovine bones at different calcination temperatures show crystallinity levels above 80%, with the highest crystallinity observed at 900°C, as presented in Table 4 .

**Table 4:** Crystallinity degree of BHA-derived Aceh bovine bones

Calcination temperature (°C)	Crystalline area fraction	Diffractogram area	Crystallinity percentage (%)
700	23080	27980	82.5
800	22807	27393	83.3
900	24288	28158	86.3
1,000	22438	26722	84.0

Abbreviation: BHA, bovine hydroxyapatite.

### SEM-EDX Characterization

**Fig. 5:**
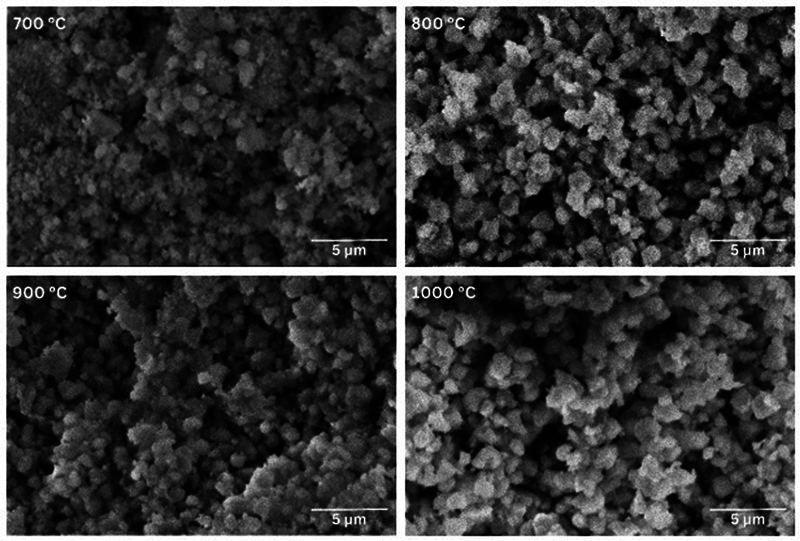
Scanning electron microscopy (SEM) micrographs of bovine hydroxyapatite (BHA) at 10,000× magnification.

**Fig. 6:**
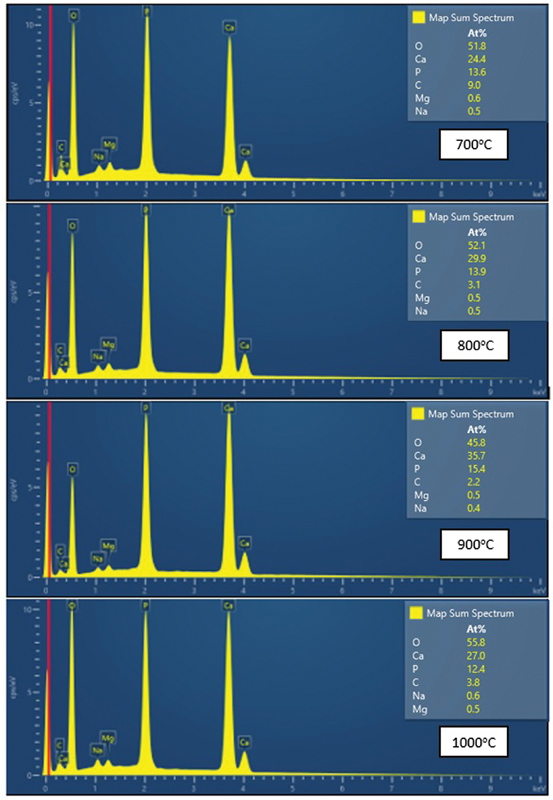
Energy dispersive X-ray (EDX) characterization of the bovine hydroxyapatite (BHA).

The morphological characteristics and elemental composition of the BHA samples at different temperatures can be observed in the SEM graphs ( Fig. 5 ). Moreover, the particles appear spherical, with some forming aggregates. The gap between particles becomes closer, and particle aggregation increases as the calcination temperature rises. Based on the chemical composition graph in Fig. 6 , BHA from calcination at different temperatures contains dominant chemical elements: calcium (Ca), oxygen (O), and phosphorus (P). Additionally, minor elements such as carbon (C), sodium (Na), and magnesium (Mg) are present. The presence of trace elements like Na and Mg, even in minimal amounts (< 1%), distinguishes BHA from synthetic HA.

EDX is an analytical technique used for elemental analysis or chemical composition of a sample. Fig. 6 represents the EDX data for Aceh bovine bone-derived BHA at 700, 800, 900, and 1,000°C, respectively. Based on the EDX results, the Ca/P weight ratio for BHA was calculated and was found to be 1.79, 2.15, 2.31, and 2.18 at 700, 800, 900, and 1,000°C, respectively ( Table 5 ). As the Ca/P weight ratio of the BHA at different temperatures did not show any considerable difference, it can be inferred that Ca/P weight ratio is independent of calcination temperature. It can be conclude that the temperature change does not significantly affect the value of Ca/P in BHA-derived calcined Aceh bovine bone.

**Table 5:** Ratio Ca/P of BHA-derived Aceh bovine bone

Temperature (°C)	At% Ca	At% P	Ratio Ca/P
700	24.4	13.6	1.79
800	29.9	13.9	2.15
900	35.7	15.4	2.31
1,000	27.0	12.4	2.18

Abbreviations: BHA, bovine hydroxyapatite; Ca, calcium; P, phosphorus.

## Discussion

This study successfully prepared BHA from bovine femur bones using calcination at varying temperatures of 700, 800, 900, and 1,000°C in a furnace. The limitation of this study was that it focused only on the characteristics of BHA obtained from the Aceh region. The cortical part of the femoral bone was utilized due to its morphological and structural similarity to human bone. 15 Based on the analysis of percentage yield, all calcination temperatures showed BHA yield percentages above 50%. These results indicate that the BHA content in Aceh bovine bones is a major component. This also demonstrates the potential use of bovine bone waste as a source of natural BHA.

The lowest percentage yield at 700°C (52.62%) suggests that BHA was not fully extracted at this temperature. Calcination temperatures of 800 and 1,000°C produced nearly identical yields of 59.56 and 59.80%, respectively. The optimal temperature for the highest BHA yield was 900°C, which resulted in the highest yield of 60.54%. Previous studies have reported that BHA synthesized at a calcination temperature of 900°C for 6 hours can achieve a yield of 83.41%. 17 BHA derived from bovine bones has shown higher yields compared with other mammalian sources, reaching 65% when extracted using calcination at 850 to 1000°C for 1 hour 18 and 72.3% at 800 to 1,000°C for 2 hours. 3

The FTIR spectra in this study indicate that the calcination process at different temperatures leads to significant changes in the chemical composition and crystal structure of the extracted BHA. The phosphate groups, which are the main peaks of HA, appear around 1,100, 1,040, and 960/cm. Absorption peaks around 1,410 and 1,450/cm indicate the presence of carbonate groups, which may result from substitution within the HA structure. The presence of carbonate groups in HA at wave numbers 1,450 and 1,990/cm was also reported by Sossa et al. 19 Additionally, Cimdina and Borodajenko reported carbonate groups at wave numbers 850 to 900, 1,300 to 1,725, and 1,950 to 2,250/cm. 20 The peak around 3,400/cm indicates the presence of hydroxyl groups, suggesting a pure HA structure. These findings are supported by previous studies, where the functional groups of HA from various bovine bones showed characteristic absorption peaks for phosphate, carbonate, and hydroxyl groups. 2 6 10 12 21

Increasing the calcination temperature changes the peak absorption intensity ( Table 2 ). The intensity of the phosphate peak tends to increase with temperature, indicating enhanced crystallinity of HA. Conversely, the intensity of the carbonate peak tends to decrease, suggesting the decomposition of carbonates at higher temperature. There is a slight shift in the absorption peak positions of the phosphate and hydroxyl groups with increasing temperature. The FTIR results in this study show that increasing the calcination temperature tends to improve peak resolution and increase crystal order, especially for the phosphate peaks.

Based on the FTIR spectra, the temperature of 900°C appears to be the best for further research. At this temperature, the phosphate peak intensity indicates good crystallinity, suggesting increased HA purity, and the hydroxyl band remains sufficiently visible. This suggests that HA has a fairly pure structure and high crystallinity at this temperature, suitable for further applications. 22 23

The particle size data presented in Table 3 shows that D50% = 38.165 µm means that 50% of the BHA sample is smaller than 38.165 µm. The D50% value is also called the “median” and divides the particle size distribution into an equal number of “smaller” and “larger” particles. Nanometer-scale biomaterials (up to 100 nm) are less effective in supporting osteoclast proliferation and bone healing due to increased osteoclast apoptosis and suppressed gene expression. In contrast, submicron-scale HA promotes osteoblast differentiation more effectively. 24 25 Studies show that nanostructured HA discs impair osteoclast formation and function, reducing cell fusion, osteoclast size, and resorption activity, while increasing apoptosis and suppressing gene expression. This emphasizes the importance of surface structure in CaP ceramics for osteoclastogenesis and their osteoinductive potential. 26 Furthermore, the size, topography, and wettability of HA nanoparticles enhance osteoblast adhesion by promoting vitronectin adsorption, supporting osteoblast function and providing insights for designing orthopedic implants that regulate both osteogenesis and osteoclastogenesis. 8 In this study, BHA particles tend to undergo sintering at higher temperatures, causing particles to merge and form larger particles. A significant agglomeration is observed at 900°C, as seen from the very large particle sizes at the 75% percentiles. It indicates that this temperature promotes the growth of smaller particles into larger ones, while increase the calcination temperature leads to an increase in particle size.

The crystallinity of Aceh cattle bone BHA showed a range of 82.5 to 86.3%, which is in a good enough range (generally 70–90%) to provide the bioactivity needed to stimulate new bone formation and encourage the process of osseointegration. A similar outcome was reported by Resmim et al, 18 where the crystallinity of BHA calcined at 1,000°C reached 84%. However, based on ISO 13779-3, it is necessary to determine foreign phases if the crystallinity is < 95% because carbonate and hydrogen phosphate phases are usually not read on XRD. Pure HA is identifiable by peaks at 202, 210, and 211, which correspond to 34, 28, and 31 at 2θ. 21 Biomaterials with a hexagonal structure, such as HA, have been reported to promote osteoblast adhesion due to their properties and performance similar to calcium phosphate compounds in the biomineralization of human mesenchymal stem cells. 26 27

As the temperature increases, the peaks become sharper and more defined, indicating a higher degree of crystallinity and more defined growth of the crystalline structure. 2 6 The presence of sharp peaks throughout the pattern confirms the crystalline nature of the material. Variations in peak positions indicate changes in lattice parameters, possibly due to thermal expansion or phase transitions. In this study, at lower temperatures (700 and 800°C), the peaks are broader and less defined, indicating smaller and less ordered crystalline structures or a higher amorphous content. At higher temperatures (900 and 1,000°C), the peaks are sharper, indicating larger grain sizes and better crystalline order. The increase in calcination temperature leads to larger crystal growth and increased crystallinity, as evidenced by SEM and XRD analyses. The increase in particle size indicated by PSA also supports these findings.

Based on the SEM-EDX result, as the calcination temperature increases, the particle size decreases, and the gaps become smaller with more uniform distribution. Qualitative analysis reveals differences in particle morphology and gap among the four BHA micrographs based on calcination temperature. 16 At all calcination temperatures, calcium and phosphorus were well distributed, maintaining a consistent chemical composition in the BHA. The micrographs of BHA at 700°C showed irregular and dispersed particles, indicating low sintering and partial crystallinity. Raising the calcination temperature to 800°C improved uniformity and shape definition, reflecting enhanced crystallinity and sintering. The micrographs of BHA at 900°C displayed clear crystalline structures and more uniform sizes, with denser particles and smaller gap like pore sizes. Calcination at this temperature leads to better sintering and potential surface diffusion processes. The micrographs of BHA by calcination at 1,000°C were morphologically similar to those at 900°C. 28 29

Overall, the results indicate that variations in sintering temperature affect the crystal size and degree of crystallinity of the samples. The sizes and shapes of synthetic BHA crystals vary with calcination temperatures, as higher temperatures promote nucleation and particle agglomeration, resulting in larger average agglomerates. As the sintering temperature increases, the energy received by the atoms is greater, allowing them to diffuse and agglomerate more, leading to larger crystal sizes and stronger, more orderly atomic bonds. 30

The Ca/P ratio of BHA from this study obtained a higher Ca/P, so it has not met the stoichiometric Ca/P ratio of 1.67, as well as the requirements of ISO 13175-3, ISO 13779-1, and ISO 13779-2 standards, namely, the molar ratio of calcium to phosphorus, Ca/P of 1.66 ≤ Ca/P ≤ 1.71. A higher Ca/P ratio than stoichiometric HA can be attributed to the presence of other calcium and phosphorus derivatives, such as CaO or CaCO _3_ , which may contribute to the overall composition of the material. 18

Natural HA is nonstoichiometric because it is usually deficient in calcium (Ca/P < 1.67) or phosphorus (Ca/P > 1.67). In cases of calcium deficiency, positions within the HA crystal structure are filled by cations such as Na ^+^ , Mg ^2+^ , or Al ^3+^ . Conversely, a lack of phosphate ions may lead to substitution by carbonate ions, which contributes to the nonstoichiometric nature of the material. 31

The carbonate content in HA is known to enhance bone integration without significantly reducing mechanical strength. A balance between biocompatibility and mechanical strength is achieved, as studies have shown that carbonate levels in the range of 5 to 10% support osteogenesis (bone formation) while maintaining the material's stability in the body. However, excessive carbonate content can reduce the mechanical strength of the material, rendering it less suitable for application as an implant that requires high mechanical properties due to its function of replacing bone. The high carbonate content in HA sourced from bovine (or other animal) bone may be due to the biological composition and natural characteristics of bone mineralization in the animal, as well as other factors such as diet, environment, and mineral metabolism patterns in bovine. 19 32 33 34

EDX results show that the primary elemental composition of BHA remains stable across various calcination temperatures, indicating that the calcination process does not significantly affect the main chemical composition of BHA. FTIR and EDX analyses reveal that the chemical composition of BHA becomes purer with increasing calcination temperature, which is reflected in the decrease of carbonates and increased order of phosphate structures. Other study has reported carbonate ions as impurities in BHA. 2 On the other hand, carbonated HA enhances osteoblast proliferation, accelerates osteoprogenitor cell differentiation, and increases collagen matrix gene expression, leading to better new bone formation and higher bone-to-material contact compared with uncarbonated HA. 24 The increased peak intensity at higher temperatures indicates higher purity or phase homogeneity of the crystalline material, with possible phase transitions suggested by the shift or appearance/disappearance of certain peaks in the XRD analysis.

Based on the characterization results using FTIR, XRD, and SEM-EDX, the four groups of BHA derived from Aceh bovine bones exhibit specific chemical, physical, and morphological properties. Chemically, they contain functional groups characteristic of HA, namely, carbonate, phosphate, and hydroxyl groups. Physically, they demonstrate moderate crystallinity, ranging from 82.5 to 86.3%, with crystal sizes of approximately 19 to 20 nm. Morphologically, the particles are spherical, with increasing particle sizes due to agglomeration. The particle density becomes closer as the calcination temperature rises, reducing the visible pore-like spaces between particles in the SEM micrographs. The average particle size of the BHA is within the submicron to micron range. However, EDX analysis reveals a Ca/P ratio ranging from 1.7 to 2.3, which is higher than the stoichiometric Ca/P ratio of human bone HA (1.67). This discrepancy arises from the presence of A-type and B-type carbonate apatite, commonly found in biogenic HA from natural sources. These carbonate apatites substitute phosphate groups in the HA structure. The presence of carbonate apatite is further confirmed by additional carbonate peaks observed in the FTIR spectrum, notably at 1,600 and 1,990/cm.

The physicochemical properties of BHA derived from Aceh bovine bones exhibit a crystallinity range (70–90%) that is considered optimal for bioactivity, enabling the stimulation of new bone formation and promoting osseointegration. The submicron-to-micron particle size enhances the interaction between the material and osteoblast cells, accelerating new bone tissue formation (bioactivity) while balancing structural stability. 24 These particles are small enough to improve bioactivity but remain stable within the body, resisting rapid degradation or dissolution. Additionally, their size supports osteoconductivity, providing a “scaffold” for new bone-forming cells. Given these characteristics, BHA from Aceh bovine bones shows potential as a bone filler or graft material for treating bone defects in dental applications. The Ca/P ratio exceeding 1.67, combined with FTIR results showing abundant carbonate ion groups, indicates the presence of carbonate apatite in the material. Naturally derived BHA often contains A-type and B-type carbonate apatites, which can enhance biodegradability and the ability to stimulate new bone growth. This makes Aceh bovine bone-derived BHA a suitable candidate for applications in hard tissue regeneration.

A carbonate content of 5 to 10% is considered optimal, as it enhances biocompatibility and closely resembles the natural composition of bone minerals, which also contain carbonate. This level supports better bone integration without significantly compromising mechanical strength. The balance between biocompatibility and mechanical stability is achieved, as most studies indicate that carbonate content within this range promotes osteogenesis (bone formation) while maintaining material stability in the body. However, higher carbonate content can reduce the mechanical strength of the material, making it less suitable for implants requiring high mechanical performance, such as load-bearing bone replacements. 19 32 33 34 35

The comparison between Aceh bovine-derived BHA, regular bovine BHA, and commercial HA reveals similar chemical characteristics, including the presence of HA-specific functional groups such as phosphate, carbonate, and hydroxyl. In terms of physical properties, Aceh bovine BHA displays a crystallinity level comparable to other bovine BHAs and commercial HA, ranging from 80 to 90%. 31 36 The crystallinity of natural BHA is typically below 95%, lower than that of pure HA, due to the presence of trace elements such as Na, Mg, and Al cations, as well as substitution groups like carbonate within the crystal structure. These substitutions also result in a Ca/P ratio exceeding 1.67. 36 37

Using specific fabrication methods and optimizing BHA extraction processes can significantly reduce the Ca/P ratio to approach the ideal value of 1.67. However, the presence of cations and substitution ions enhances the bioactivity and osteoconductivity of the material. Particle size in BHA is heavily influenced by the fabrication method; calcined BHA that undergoes grinding, sieving, and ball milling typically produces submicron-to-micron-sized particles.

This preliminary study, focused on characterization, concludes that the physicochemical properties of Aceh bovine BHA show potential as a raw material for synthesizing bone filler biomaterials for medical and dental applications. However, its suitability as an implant material for orthopaedic, dental, and maxillofacial applications according to ISO 13779 standards for HA in implant surgery has not yet been confirmed. Further improvements in BHA properties are needed through optimized extraction and synthesis methods. Additional testing, including solubility, mechanical properties, and bioactivity assessments, is essential to validate its applicability.

## Conclusion

This study contributes new insights into the field of BHA research by demonstrating that BHA can be effectively synthesized from Aceh bovine femur bones through calcination at temperatures ranging from 700 to 1,000°C. FTIR and XRD characterization confirm that the primary phase of the material is BHA, with a crystallinity exceeding 80%. The analysis also identifies the presence of functional groups, including phosphate, carbonate, and hydroxyl groups. Increasing the calcination temperature results in BHA with higher crystallinity, larger particle size, and improved chemical purity. Based on the characterization results, calcination temperatures of 900 and 1,000°C appear to provide optimal outcomes in terms of crystallinity and uniform particle size. However, the final temperature choice for further applications may depend on specific requirements, such as the desired particle size or mechanical properties. This study is not without its limitations. Future research should explore the economic and environmental advantages of repurposing bovine bone waste as a sustainable source of HA. Such an approach could significantly reduce waste and production costs, particularly for dental and biomedical applications. Additionally, further investigations into the mechanical properties, biocompatibility, and long-term performance of the synthesized BHA in clinical settings will be essential to ensure its practical applicability. Despite its contributions, this study is not without limitations, which future studies should aim to address.
